# Insights into the Influence of Specific Splicing Events on the Structural Organization of *LRRK2*

**DOI:** 10.3390/ijms19092784

**Published:** 2018-09-16

**Authors:** Dimitrios Vlachakis, Nikolaos E. Labrou, Costas Iliopoulos, John Hardy, Patrick A. Lewis, Hardy Rideout, Daniah Trabzuni

**Affiliations:** 1 Genetics Laboratory, Department of Biotechnology, Agricultural University of Athens, 75 Iera Odos Street, 11855 Athens, Greece; dimvl@aua.gr; 2 Laboratory of Enzyme Technology, Department of Biotechnology, School of Food, Biotechnology and Development, Agricultural University of Athens, 75 Iera Odos Street, 11855 Athens, Greece; lambrou@aua.gr; 3 Department of Informatics, Faculty of Natural and Mathematical Sciences, King’s College London, Strand, London WC2R 2LS, UK; csi@kcl.ac.uk; 4 Department of Neurodegenerative disease, UCL Institute of Neurology, Queen Square, London WC1N 3BG, UK; j.hardy@ucl.ac.uk (J.H.); p.a.lewis@reading.ac.uk (P.A.L.); 5 School of Pharmacy, University of Reading, Whiteknights, Reading RG6 6AP, UK; 6 Division of Basic Neurosciences; Biomedical Research Foundation of the Academy of Athens, Soranou Efessiou 4, 11527 Athens, Greece; hrideout@bioacademy.gr; 7 Department of Genetics, King Faisal Specialist Hospital and Research Centre, Riyadh 11211, Saudi Arabia

**Keywords:** LRRK2 mRNA expression, Human brain substantia nigra, Parkinson’s disease (PD), ROC/COR domain splicing events, WD40 domain in protein structure

## Abstract

Leucine-rich repeat kinase 2 (LRRK2) is a large protein of unclear function. Rare mutations in the *LRRK2* gene cause familial Parkinson’s disease (PD) and inflammatory bowel disease. Genome-wide association studies (GWAS) have revealed significant association of the abovementioned diseases at the *LRRK2* locus. Cell and systems biology research has led to potential roles that LRRK2 may have in PD pathogenesis, especially the kinase domain (KIN). Previous human expression studies showed evidence of mRNA expression and splicing patterns that may contribute to our understanding of the function of LRRK2. In this work, we investigate and identified significant regional differences in LRRK2 expression at the mRNA level, including a number of splicing events in the Ras of complex protein (Roc) and C-terminal of Roc domain (COR) of LRRK2, in the substantia nigra (SN) and occipital cortex (OCTX). Our findings indicate that the predominant form of LRRK2 mRNA is full length, with shorter isoforms present at a lower copy number. Our molecular modelling study suggests that splicing events in the ROC/COR domains will have major consequences on the enzymatic function and dimer formation of LRRK2. The implications of these are highly relevant to the broader effort to understand the biology and physiological functions of LRRK2, and to better characterize the role(s) of *LRRK2* in the underlying mechanism leading to PD.

## 1. Introduction

Non-synonymous coding mutations in the leucine-rich repeat kinase 2 (*LRRK2*) gene on chromosome 12p12 were identified as a cause of autosomal dominant Parkinson’s disease (PD) in 2004 [1,2]. Since then, an extensive amount of research has been conducted to identify the function and dysfunction, activity, and localization of this protein. The complex domain architecture of LRRK2, shared with its paralog LRRK1, provides membership in two distinct protein families: The ROCO family, and the receptor-interacting protein (RIP) kinase family [3]. The ROCO family proteins are characterized by their tandem Ras of complex proteins (ROC)/GTPase domain, followed by the C-terminal of ROC (COR), domains. In humans, there are 4 members of this family, LRRK1, LRRK2, death associated protein kinase 1 (DAPK1), and malignant fibrous histiocytoma-amplified sequence 1 (MFHAS1, also known as MASL1) [4]. The human RIP kinase family has seven known members, distinguished by common and functionally overlapping interacting partners and signalling pathways, linked to the plasma membrane “death receptors” and the NF-κB network [5]. LRRK2 has been implicated in a wide range of physiological processes, including neurite growth, vesicle trafficking, cytoskeletal maintenance and autophagic protein degradation [3]. Additional genetic studies, most notably genome-wide association studies (GWAS) have revealed the association of variants at the *LRRK2* locus with inflammatory bowel disease, Leprosy and PD [6,7,8,9,10].

*LRRK2* is a large gene located on chromosome 12p12 consisting of 51 exons. The golden Havana/Ensembl database reports a total of seven transcripts for the *LRRK2* locus, four of which are protein-coding; 2 with retained introns; one processed transcript and one nonsense-mediated decay. The longest, full-length transcript encodes a 2527 amino acid protein [11,12]. Until recently, very little is known about the mRNA expression and splicing patterns of LRRK2 in human tissues. In a previous study, we investigated the expression and splicing data of LRRK2 from 134 healthy control individuals in 10 human brain regions (Braineac dataset, UK brain expression consortium (UKBEC)) [13,14]. Microarray analysis of *LRRK2* gene mRNA expression revealed evidence of regional mRNA transcript variability. Specifically, expression profiling of the LRRK2 transcript demonstrated a 2.4 fold change (FC), (*p* value = 2.7 × 10^−51^) across the analysed brain regions, with the occipital cortex (OCTX) showing the highest expression, while cerebellum (CRBL) showed the lowest expression. Importantly, this study also provided evidence of significant splicing events existing in different brain regions, especially in OCTX and SN. Furthermore, expression and exon Quantitative Trait Loci (QTLs) were reported in this study, highlighting that splicing pattern observed for the *LRRK2* gene is under genetic control [13]. An exon/splicing QTLs (sQTL) was identified within exon 32 and 33, indicating a possible relevance in splicing in this region. The sQTLs associated with an increase of the expression of exon 32 and 33, showed the highest significance (FDR ranging from 2.70 × 10^−7^ to 6.47 × 10^−2^) in the CRBL, medulla and white matter regions. These results motivated us to translate and model these splicing events at the structural level to better understand the functional consequences.

From a structural perspective, a crystal structure for the human ROC/GTPase domain was elucidated in 2008 [15], and a number of structures for the ROC/COR and KIN domains have been reported for orthologous proteins from other organisms [16,17]. Further studies have utilized homology modelling and in silico docking studies using proteins with similar domains and domain architectures as templates. In a recent example, a model for full-length LRRK2 derived from electron microscopy (EM) data, inter-domain interaction data from mass spectrometry, and homology modelling was proposed by Guaitoli and colleagues [18]. The resulting 3-dimensional model for full-length dimeric LRRK2 revealed close conformational positions between relatively distant domains, such as the C-terminal kinase domain and N-terminal ankyrin repeats. Importantly, although the final model presented was based upon the overall topology of purified LRRK2 particles revealed by negative stain EM, and further constrained by cross-linking, there was evidence for marked variability in the 3-dimensional organisation of the LRRK2 particles observed, an indication that multiple configurations of dimeric LRRK2 are possible. This is likely to be accentuated by the wide variety of putative binding partners identified thus far [19].

In this current study, we report evidence supporting the existence of multiple LRRK2 splice variants (removal of exons 32–33 [ROC-COR], exons 42–43 [kinase (KIN)] and exons 48–50 [WD40]) within multiple brain regions, and model the projected structural implications of LRRK2 splice variants on the resulting protein.

## 2. Results

**Figure 1:**
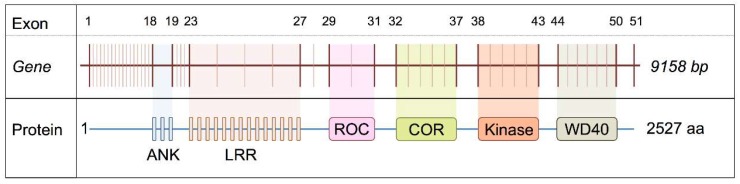
Schematic view shows the physical position for certain exons corresponding to certain functional domains in *LRRK2* gene.

Based on our previous identification of differences in LRRK2 mRNA exon abundance in targeted regions between different regions, of the human brain [13] we set out to test whether these are, due to altered splicing of LRRK2 transcripts. A summary of the relationship between exon number and the domains of *LRRK2* gene is shown in Figure 1.

**Figure 2:**
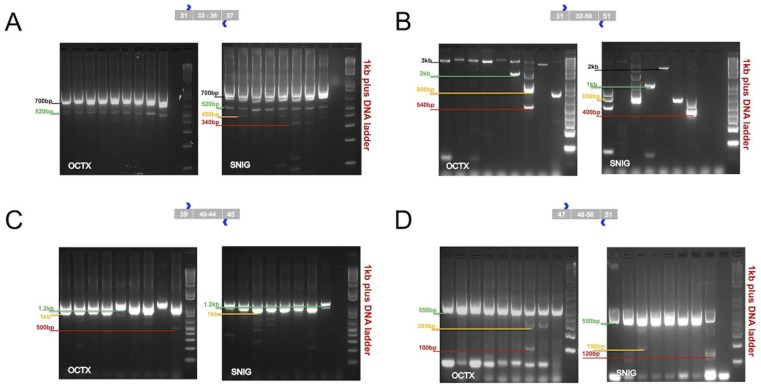
(**A**) RT-PCR results confirming differential splicing in targeted region (exon 32–36) corresponding to functional ROC-COR domains in *LRRK2* gene in occipital cortex (OCTX) and substantia nigra (SN) (SNIG). It is clear the two regions have different patterns from same nine individuals for OCTX and eight individuals for SN. The expected band for this primer pair is ~870 bp, in OCTX 2 bands at ~520 and 700 bp are shown. In SN more bands are shown, one at 340, 400, 520 and 700 bp; (**B**) RT-PCR results confirming differential splicing in targeted region (exons 32–50), corresponding to the functional COR-KIN-WD40 domains in *LRRK2* gene in OCTX and SN. The expected band for this primer pair is ~3 kb, in OCTX the expected band is shown, in addition to other bands ~540, 900 and 2 kb. SN has different bands, at 400, 650, 1 kb, 2 kb and more; (**C**) RT-PCR results confirming differential splicing in targeted region (exons 40–44). It is corresponding to the kinase domain in the *LRRK2* gene in OCTX and SN. The expected band for this primer pair is ~1 kb, in OCTX the expected band is shown, in addition to other bands ~500 and 1.2 kb. SN has similar bands, at 500, 1 kb (expected band), 1.2 kb and more; (**D**) RT-PCR results confirming differential splicing in targeted region (exons 48–50). It is corresponding to the WD40/C-terminus domains in the *LRRK2* gene in OCTX and SN. The expected band for this primer pair is ~550 bp, in OCTX the expected band is shown, in addition to other bands ~100 and 300 bp. SN has similar bands, at 120, 150 and 550 bp (expected band) and more.

Reverse transcriptase polymerase chain reaction (RT-PCR) experiments were performed to cover the following regions: (A) Ras-like G-domain (Roc)- C-terminal of Roc domain (COR) (exons 32–36, 870 bp), (B) COR-kinaseKIN-WD40 (exons 32–50, 3 kb), (C) Kinase domain (KIN) (exons 40–44, 1 kb), and (D) WD40/C-terminus (exons 48–50, 550 bp) domains. These domains play a fundamental role in the enzymatic function of LRRK2 protein. This characterization was applied to confirm and define mRNA splicing patterns of the LRRK2 present in the SN and OCTX and to understand the impact of these splicing events on the predicted protein product. These two regions were selected, because SN is an important brain area to investigate in some neurodegenerative diseases, particularly in PD, while, OCTX showed the highest expression level of LRRK2 between the other 10 brain regions in our previous microarrays study [13]. Both regions represented the splicing events in the microarray data [13]. In the ROC-COR region (encoded by exons 30–36), in addition to the expected band of 870 bp, other bands of sizes were approximately 520 and 700 bp (in OCTX) and 340, 400, 520 and 700 bp (in SN) were observed (Figure 2A). Similarly, for the COR-KIN-WD40 region, spanning exons 32–50, in addition to the primary expected fragment (3 kb), other bands of sizes 540 bp, 900 bp and 2 kb (OCTX), and 400 bp, 650 bp, 1 kb, and 2 kb (SN) were amplified (Figure 2B).

For the kinase domain (encoded by exons 40–44), in addition to the primarily expected fragment (3 kb), additional bands at sizes of 500 bp and 1.2 kb were observed and targeted in OCTX and SN (Figure 2C). Exon 41 encodes part of the kinase domain containing the G2019 and I2020 residues, both of which are mutated in familial forms of PD (to a serine and threonine respectively). For the last domain, the WD40/C-terminus (encoded by exons 48–50), in addition to the expected band of 550 bp, other bands at sizes of 100 and 300 bp (in OCTX), and 120 and 150 bp (in SN) were detected (Figure 2D).

In summary, our RT-PCR results support the existence of smaller fragments suggesting complex exon splicing (exclusion) events spanning different domains of LRRK2. Minor splice isoforms exist, albeit in low abundance; however, our findings indicate that the majority of LRRK2 transcripts in these tissues are likely to be full length. It is important to note that these data do not exclude the possibility of other mRNA fragments that were not amplified under these conditions. Nor do our findings rule out the possibility of specific splice isoforms occurring in greater abundance in *LRRK2* mutation carriers.

Different specific targeted bands in addition to lower sized bands were obtained, amplified, extracted and purified followed by nested PCR reactions. Nested PCR was performed in order to amplify the low-intensity bands of the minor isoforms, followed subsequently by direct Sanger sequencing. Different PCR product clean-up protocols were applied to the nested PCR products before the sequencing reactions to increase the likelihood of obtaining high-quality sequencing reads from low the abundance isoforms. Sanger sequencing data were analyzed by using the Sequencer DNA sequence alignment and analysis software Version 5. Unfortunately, only one sample in the forward direction was of sufficient quality to be aligned and read in comparison to the reference sequence.

RNA sequencing, as an additional technique to capture and confirm the minor isoforms, as well as to identify the splicing junctions at low abundance for the region of ROC-COR (exons 32–36) was recently used [13]. In this study, four OCTX and four CRBL samples were assessed. Results revealed a minority of exon 31–34 exon-exon junction reads (from 3 to 5 reads) and 14 reads for exon 31–33 exon-exon junctions out of 430 reads for the exon-exon junction of exon 31–32; in comparison to other exon-exon junctions along the transcript. In addition, the ability to detect expression of the minor isoforms, in contrast to the major isoforms, was significantly reduced; likely due to the small sample size. and we believe this reflects the complexity of splicing events, particularly for a gene of the size of *LRRK2* gene.

After confirming the corresponding splicing patterns to specific functional LRRK2 domains in this report, the 3D structures of the ROC-COR, kinase (KIN), and WD40 domains of LRRK2 were modelled in an effort to structurally assess the effect of three splicing events. As described above, splicing event 1 involves the removal of exons 32–33; the second involves the removal of exons 42–43 respectively, and the final splicing event modelled here affected the WD40 domain, exons 48–50. The removed exons 32–33 correspond to the loss of the following amino acid sequence located principally within the COR domain: “^1513^IRDQLVVGQLIPDCYVELEKIILSERKNVPIEFPVIDRKRLLQLVRENQLQLDENELPHAVHFLNESGVLLHFQDPALQLSDLYFVEPKWLCKIMAQ^1609^”; whereas the removed exons 42–43 correspond to the loss of the sequence: “^2037^GFRAPEVARGNVIYNQQADVYSFGLLLYDILTTGGRIVEGLKFPNEFDELEIQGKLPDPVKEYGCAPWPMVEKLIKQCLKENPQERPTSAQ^2127^”, located within the kinase domain, spanning sub-domains 8–11. Splicing event 1 accounts for the loss of a total of 97 amino acids, while splicing event 2 involves the loss of 91 amino acids. The splicing of exons 48–50 corresponds to the loss of 144 amino acids between residues ^2343^FSYAAFSDSNIITVVVDTALYIAKQNSPVVEVWDKKTEKLCGLIDCVHFLREVMVKENKESKHKMSYSGRVKTLCLQKNTALWIGTGGGHILLLDLSTRRLIRVIYNFCNSVRVMMTAQLGSLKNVMLVLGYNRKNTEGTQKQK^2487^.

**Figure 3:**
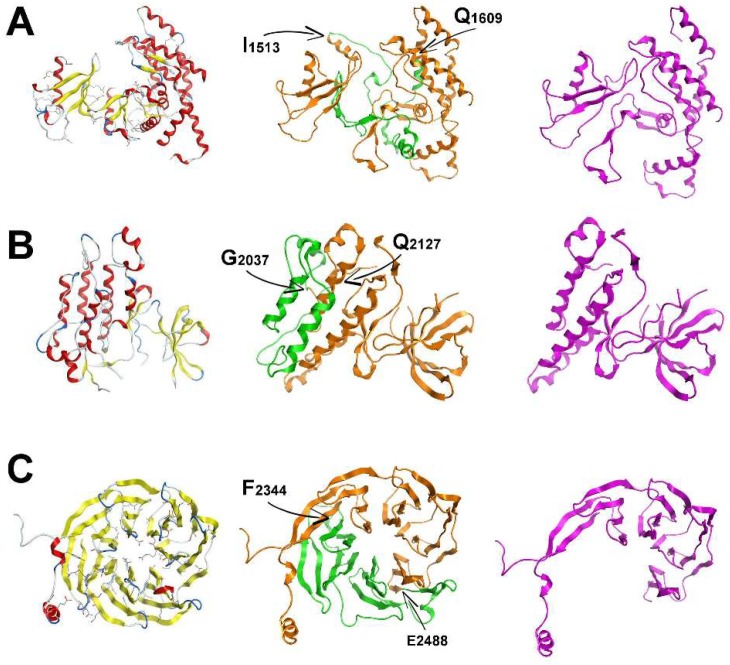
A molecular modelling study of the COR, KIN and WD40 domains of LRRK2 (**A**, **B**, and **C** respectively). In the left column is the full 3D homology model of each one of the three aforementioned domains of LRRK2. They are shown in ribbon representation and are color-coded by secondary elements, where α-helices are red spirals, β-sheets are yellow arrows and loops are white coils. The middle column depicts the same three 3D homology models as in the left column. Models have also been rendered in ribbon representation, only this time they have been colored based on their corresponding splicing events. The orange color is the full model, whereas in the green ribbon is the part of the structure that is absent in the spliced form. The start and end points of each splicing event have been highlighted on the 3D structure of the three models. Finally, the third column contains the spliced 3D models of ROC, COR and WD40, and is shown in purple ribbon representation. Note that in the case of COR, the spliced 3D model had to be stitched together as it is a mid-segment of the structure that is spliced, whereas in the cases of KIN and WD40 the part of the structure was removed, and the spliced structures were in silico energetically re-optimized.

The 3D structure for the COR domain of LRRK2 suggests that it can tolerate the potential removal of exons 32–33 as it is comprised of two large regions that are linked by a flexible stretch of two structurally weak antiparallel beta-sheets (Figure 3A), which are lost with the removal of exons 32–33 (Figure 3A). As a result, the molecular dynamics simulation of the spliced molecular system confirmed a more robust and compact conformation for the spliced ROC domain of LRRK2 (Figure 3A). The resulting shortened COR domain has lost the ‘hinge’ properties of the wild-type COR and the rather flexible interconnecting beta-sheets (Figure 3A). We predict that this may result in locally increased structural robustness that may have an effect on the overall folding of LRRK2, and likely its dimer formation.

The KIN 3D model of LRRK2 is also made up of two distinct domains. One is a relatively larger network of alpha-helices and the smaller one is a set of interacting beta-sheets. The larger sub-domain within the KIN model consists of four primary alpha-helices that construct an antiparallel alpha-helix bundle that is also supported by satellite shorter alpha-helices. Upon splicing of exons 42–43, two of the four above-mentioned alpha-helices are lost and therefore the alpha helix bundle is disrupted (Figure 3). Of interest, the hydrophobic core of the original four-helix bundle is now exposed to solvent, which is predicted to have a destabilizing effect on the overall folding of the spliced KIN domain of LRRK2. The LRRK2 protein with the novel spliced KIN domain is more compact and smaller in size, but with a set of two antiparallel alpha-helices; and exposed to solvent the intra helix hydrophobic interface. Based on these models, we hypothesize that this splicing event is used as an interaction switch by LRRK2 as the resulting protein with spliced KIN domain is optimally folded to accept a protein partner with the complementary two antiparallel missing alpha-helices to re-construct the original four-helix bundle and secure the hydrophobic intra-helix interface. Further studies examining this possibility are underway.

The model of the LRRK2 WD40 domain is comprised of a set of 28 antiparallel β-sheets in a barrel-like formation (Figure 3). The β-sheets are interconnected via a consistent network of hydrogen bonds and hydrophobic interactions. The 28 β-sheets are grouped in β-sheet bundles of four β-sheets each. Each one of the seven β-sheet bundles is linked to the next one via a flexible hairpin loop. The c-terminal loop has adopted a α-helical conformation of two and a half spirals. The region that is spliced is the part of the template structure that interacts with a co-crystallized α-helix. The coordinates of that α-helix were transferred to the model and it was confirmed that the interacting portion of WD40 falls exactly in the spliced region (Figure 3). There is a set of specific hydrogen bonds (mainly between K/R–D/E residues) that secures the interaction (Figure S1). The anatomy and 3D conformational arrangement of the interacting α-helix that was transferred from the eukaryotic translation initiation factor eIF3i complex with eIF3b C-terminus eIF3i (Protein Data Bank (PDB) ID:3ZWL) crystal structure is in good agreement with the conformational arrangement of the modelled WD40 structure that is spliced (Figure S2).

## 3. Materials and Methods

### 3.1. Human Post-Mortem Brain Tissues Collection

The *Substania nigra* (SN) and occipital cortex (OCTX) samples from 10 individuals were selected as a subset of this focused study from the full UK brain expression consortium (UKBEC) cohort. The brain tissues originating from 137 control individuals were collected by the Medical Research Council (MRC) Sudden Death Brain and Tissue Bank, Edinburgh, UK [20]. All samples had fully informed consent for retrieval and were authorized for ethically approved scientific investigation (Research Ethics Committee number 16/ES/0084, 5 June 2017; The National Hospital for Neurology and Neurosurgery & Institute of Neurology Joint Research Ethics Committee). These are male brains with age range from 16 to 62 years with average of 42 years old and post-mortem interval (PMI) ranging from 38 to 95 h; with average of 62 h. Detailed phenotypic information for each sample is described and published in more details in Trabzuni et al. [14].

### 3.2. RNA Isolation and Processing

Total RNA was isolated from human post-mortem brain tissues using a single-step method of RNA isolation [21,22] with the miRNeasy 96 kit (Qiagen, Manchester, UK). The quality of total RNA was evaluated by the 2100 Bioanalyzer (Agilent, Cheshire, UK) and RNA 6000 Nano Kit (Agilent). Further details regarding RNA isolation, quality control and processing are reported in Reference [14].

### 3.3. Semi-Quantitative Reverse-Transcriptase RT-PCR

In order to investigate the splicing events in the brain tissues and to follow up the published preliminary results from Trabzuni et al. [13], QIAGEN Long Range 2step RT-PCR kit (Qiagen, Manchester, UK) and iScript™ cDNA Synthesis Kit (BIO-RAD, Hertfordshire, UK) were used to generate cDNA. Random primers and gene-specific designed primers were used to enhance the targeting and the amplification of the interested regions. All gene-specific primers for this analysis were designed using the Primer3 software (fokker.wi.mit.edu/primer3/input.htm) and were then Basic Local Alignment Search Tool BLAST searched against University of California, Santa Cruz UCSC human in-silico PCR tools.

cDNA was synthesized from 1–2 µg of total RNA from 10 samples, originating from two brain regions (SN and OCTX) using random and gene-specific designed primers in a total reaction volume of 30 µL under the following conditions: Incubation at 42 °C for 50–90 min, followed by enzyme activation at 85 °C for 5 min, then stored at 4 °C or −20 °C ready for the next step. 2.5 µL of cDNA was used to perform the semi-quantitative RT-PCR for the targeted exons in a total reaction volume of 25 µL under the following conditions: Initial activation at 95 °C for 5 min, followed by denaturation at 94 °C for 35 s, annealing 60 °C for 55 s, elongation 72 °C for 3 min for 40 cycles. PCR products were run on different percentages of low-melt agarose gels (1.5, 2 and 3%) (Invitrogen, Loughborough, UK) in modified Tris-acetate-Ethylenediaminetetraacetic Acid(EDTA) (TAE) buffer in cold room (4 °C), and photographed using UV transilluminator to visualize GelRed staining. Images were captured and saved using Gel-Doc (BIO-RAD). At this stage, after excluding bad quality samples, we have 9 individuals from OCTX and 8 individuals from SN to continue with for these experiments.

### 3.4. PCR Primers Used in This Study

The PCR primers used in this study are summarized in Table S1.

### 3.5. DNA Extraction from Low-Melt Agarose Gel and SANGER Sequencing

After DNA fragments were run and separated by agarose gel electrophoresis, individual DNA bands of interest were sliced using a long-wavelength UV lamp transilluminator and extracted for Sanger sequencing. Based on the weight of the gel slice, different gel extraction kits were used to maximize DNA recovery. For the intense bands weighing >120 mg to 400 mg, Minelute gel extraction kit (Qiagen, UK) was used. For less intense bands, which present minor expressed isoforms, gel slices weighted between 50 to 120 mg and the Millipore DNA gel extraction kit (Montage, Millipore, Watford, UK) was used. Protocols were applied according to the manufacturer’s manual. Except at the precipitation step, the Ethanol was used for DNA precipitation instead of propanol as the kit recommended protocol. For DNA sample concentration less than 50 ng/μL; the DNA was concentrated to 70 ng/μL using Glycogen -Sodium acetate (3 M, NaOAc) (Sigma, Dorset, UK). For each 20 μL of DNA solution 1 μL glycogen + 2 μL 3 M NaOAc +75 μL ethanol were added. Samples were incubated at −20 °C overnight. Next day, samples were centrifuged for 60 min at maximum speed (16,000 *g*), the pellet was washed with 70% ethanol twice and air dried. DNAs were suspended in 15 μL TE buffer. The concentration and purity of each DNA sample were assessed by measuring its optical density (OD) at a wavelength of 260 nm, using the NanoDrop ND-1000 Spectrophotometer V3.3.0. The concentration of each sample was calculated, together with the ratio of absorbance at 260 nm/280 nm and 260 nm/230 nm. Samples with 260 nm/280 nm ratio lower than 1.7 were re-extracted from a fresh tissue block. Samples were then sent to (Source BioScience, Nottingham, UK) for standard Sanger sequencing services. For fragments larger than 600 bp (700 bp to 3 kb) additional pairs of primers were designed to overlap and cover the whole fragments. This was implemented into the original protocol to enhance the quality of the Sanger sequencing reads.

### 3.6. Molecular Modelling

All calculations and visual constructions were performed on a cluster supercomputer, using Molecular Operating Environment (MOE) version 2013.08 software package [23], developed by Chemical Computing Group (Montreal, QC, Canada).

### 3.7. Homology Modelling

The homology modeling of the LRRK2 ROC and COR domains were carried out using MOE. The selection of template crystal structures for homology modelling was based on the primary sequence identity and the crystal resolution. The model of splice variant 1, missing exons 32–33, was based on the crystal structure of human choline kinase alpha (PDB ID: 3F2R). The model of splice variant 2, missing exons 42–43, was based on the crystal structure of the Tie2 kinase domain (PDB ID: 2WQB). Finally, for the model of splice variant 3, missing exons 48–50, the crystal structures of both the eukaryotic translation initiation factor eIF3i (PDB ID:3ZWL) and PAF-AH holoenzyme-chain C (PDB ID:1VYH) were selected and threaded together to produce a hybrid template structure. The sequence alignment only marginally allowed for conventional homology modelling to be considered as sequence identity and similarity was calculated to 27% and 43% for splice variant 1, 33% and 56% for splice variant 2, respectively. The sequence identity and similarity of splice variant 3 were in the lower regions of 15–20% identity, and nearly 30–40% similarity.

The MOE homology model method is separated into four main steps. The first step is the initial partial geometry specification, where an initial partial geometry for each target sequence is copied from regions of one or more template chains. Secondly, the insertions and deletions task, where residues that still have no assigned bac kbone coordinates are modelled. Those residues may be in loops (insertions in the model with respect to the template), they may be out gaps (residues in a model sequence, which are aligned before the C-terminus or after the N-terminus of its template) or deletions (regions where the template has an insertion with respect to the model). The third step is the loop selection and side-chain packing, where a collection of independent models is created. The last step involves the final model selection and refinement, where the final models are scored and ranked after they have been stereochemically checked.

### 3.8. Model Optimization

Energy minimization was performed in MOE initially using the Amber99 force-field implemented into the same package until a root mean square deviation (RMSd) gradient of 0.0001 to remove the geometrical strain. The models were subsequently solvated with simple point charge (SPC) water using the truncated octahedron box extending to 7Å from the model. Molecular dynamics was performed at 300 K, 1 atm, with 2-second step size for a total of ten nanoseconds, using the NVT (Number of atoms, Volume and Temperature, held constant throughout the calculation) ensemble in a canonical environment. The results of the molecular dynamics simulation were collected into a database by MOE for further analysis. The produced models were initially evaluated within the MOE package by a residue packing quality function, which depends on the number of buried non-polar side-chain groups and on hydrogen bonding.

## 4. Discussion

The evidence presented here supports the central hypothesis of our study, that multiple splice isoforms of LRRK2 are present in the human brain at different ratios. We have successfully identified splicing events occurring, on a regional basis, in the substantia nigra and occipital cortex. The implications of this are highly relevant to the understanding of the physiological role of LRRK2 in the brain and to efforts to target LRRK2 in Parkinson’s disease. In particular, the validation of splicing events in the ROC/COR and KIN domains of LRRK2 raises the possibility that there are important consequences for the enzymatic function of the LRRK2 complex in these regions. To test these consequences will require the generation of recombinant fragments of LRRK2 encoding these alternate versions, and assessing the function of the truncated variant through assays predicted to selectively evaluate ROC/COR/KIN-dependent activity (e.g., GTP binding and hydrolysis, dimer formation, cellular localization, and kinase activity). We have not yet elucidated the full open reading frames of the splice variants that are represented in these regions, as these studies are currently underway.

Previous work in over-expression models has shown that the loss of the WD40 domain results in almost complete ablation of kinase activity from the full-length protein—with the G2385R risk variant in this domain also reported to significantly reduce kinase activity [24,25]. Additionally, loss of this domain affects the dimerization of LRRK2 and prevents neuronal death caused by pathogenic PD mutations [24]. It is therefore crucial when analyzing alternative isoforms from the human brain that any splicing events impacting on this domain are assessed in the context of a physiologically relevant ORF of *LRRK2*.

The three splice variants modelled here affect key regions within LRRK2 that are critical for its function. Namely, they affect the COR domain, the KIN domain, the WD40 domain, which is a key protein/protein interacting region and the location where a PD risk factor is found in East Asian populations, G2385R [26]. The COR and KIN domain splicing variants occur within the defining ROCO region of LRRK2 do not affect residues with known linkage to PD. Nevertheless, the impact of the loss of these regions on overall LRRK2 function is expected to be significant. For example, the COR domain is predicted to be crucial for LRRK2 dimer formation [17,27], which in turn facilitates kinase activity [28,29]. Current evidence indicates that LRRK2 functions as a GAD-type GTPase, which are G-proteins that are activated by nucleotide-dependent dimerization, rather than strictly dependent on the presence of GEF’s (guanine exchange factors) or GAP’s (GTPase activating proteins) [16,30]. In this form, residues within the COR domain stabilize the LRRK2 dimer, and the proximity of the two adjacent ROC domains facilitate GTP hydrolysis. It is not known, however, if specific residues within the COR domain that are necessary for LRRK2 dimer formation are present within the spliced region modelled here. However, our structural model of the isolated COR region would suggest that some inherent flexibility within this region is lost, which may impact the ability of LRRK2 to adjust to the presence of a second LRRK2 molecule. We are currently examining the formation and stability of LRRK2 dimers in the presence or absence of these residues in vitro.

Independently of the second splice variant modelled here, it is expected that the splicing of these residues within the COR domain, particularly if dimer formation is significantly impacted, will also affect LRRK2 kinase activity. Multiple reports now suggest that the bulk of total cellular LRRK2 kinase activity resides within dimeric LRRK2 [28,29], possibly concentrated at the cellular membrane [29], however, this may be cell-type dependent. Thus, if LRRK2 dimers are de-stabilized in these variants, LRRK2 kinase activity may be compromised. However, given the complexity of the domain architecture of LRRK2, particularly the existence of multiple protein interaction repeats, it is possible that such kinase-deficient variants of LRRK2 could assume other scaffolding-based cellular functions.

In most kinases, the region spanning the Mg^2+^ binding “DFG” (“DYG” in LRRK2) and the Subdomain VIII “APE” motifs make up the activation segment. In the case of the second splice variant modelled here, the splicing of exon 42–43, the C-terminal end of the activation segment containing the “APE” motif is removed. The kinase activity of this spliced variant would almost certainly be abolished. What is unclear is whether other LRRK2 cellular functions, such as GTP hydrolysis, or protein localization and interactions, would also be similarly disrupted. It is also possible that the various splice variants are still able to form protein dimers with full-length LRRK2, possibly leading to the recruitment of novel protein substrates that could be potentially phosphorylated by the intact partner. A complete understanding of the frequency of these splice variants, their co-existence with full-length un-modified LRRK2 transcript, and co-occurrence with disease-linked mutations will help resolve these outstanding questions. Further, it is difficult to predict with absolute certainty, based on the models established here, whether the LRRK2 protein would withstand the loss of these residues without becoming unstable. Our models do suggest that the structural impact of the splicing of these regions would be generally tolerated, at least within the isolated domain. However, the functional consequences would clearly be significant.

Lastly, in the third WD40 domain splice variant the α-helix belongs to the complex between the yeast seven-bladed β-propeller eIF3i/TIF34 and a C-terminal α-helix of eIF3b/PRT1 [31]. The authors report that structurally, the association pattern between those two proteins reveals universally conserved interactions. Mutation studies in this interaction region have confirmed that reduced or loss of interaction causes severe growth defects and eliminates association of eIF3i/TIF34, as well as eIF3g/TIF35 with eIF3 and 40S subunits in vivo [31]. The predicted WD40 spliced region is likely associated with this regulatory mechanism, due to its unique structural similarity [31]. The region that is lost is precisely the region that interacts with the adjacent α-helix. Consequently, we propose that this splicing event may serve as a switch that regulates the interaction of WD40 domain to the mRNA initiation machinery in a similar fashion to that reported between eIF3i/TIF34 and eIF3b/PRT1.

There are two key natural extensions of this work. First, following part of our efforts to generate sequence data for the splice junctions resulting from the alternative splicing events identified in this study, it is essential to generate sequences relating to entire isoforms, resulting in full ORF for each variant. Optimizing and obtaining high-quality full sequencing reads was challenging, as the fragments were of quite low abundance in the RNA samples from human postmortem brain tissues. This is especially problematic when targeting a long fragment of a minor isoform. These observations confirming the presence of different mRNA isoforms at different ratios point to an important sign of a specific complex pattern of the splicing events that affected and shifted the open reading frame (ORF) in a way that might not be detected by the primer design and the downstream Sanger sequencing we used in these experiments.

Secondly, functional analyses of the resulting transcript variants are necessary to understand the biological consequences of the splicing events in a cellular context, as well as different in vitro assays. As discussed previously, correlation of the observed transcript variants with endogenous protein fragments will be challenging, due in part to the technical limitation of available antibodies mapping to multiple epitopes of the protein, as well as the predicted low abundance of these variants. During this work, an effort was made to correlate transcript sequencing to amino acid sequencing in samples isolated from the human brain. To achieve this, we set out to validate immunoprecipitation protocols using the MJFF-produced anti-LRRK2 antibodies (characterized in detail in Reference [32]). To date we have been able to enrich LRRK2 as assessed by immunoblot, however, we have not been able to do so to the point where the protein is abundant enough for mass spec analysis. We are continuing to work towards this aim to achieve optimal conditions prior to attempting immunoprecipitation of truncated LRRK2 from brain material.

Additional essential questions to be resolved include the regulation of such splicing events. While multiple splice fragments were detected and amplified across different regions, including the SN, a region critical for PD pathogenesis; the context in which they are generated needs to be defined, as well as their relative abundance and contribution in a disease context. It is important to keep in mind that we have a possibility of false negatives for low-abundance fragments were not amplified under the used conditions, thus, these fragments cannot be excluded. Furthermore, it should be noted that the patterns of the amplified bands vary between the two regions and in different individuals, which reflect the brain region specificity and the effect of individual genetic background as a factor that may alter the splicing mechanism, especially in SN. The present study is underpowered to determine whether this variation is, due to inherent genetic differences between individuals, or due to the specific environment of the SN in these individuals immediately prior to death. The genetic background of all samples in the UKBEC have been determined by SNP microarray, however; genetic determination of splicing events in this region as a cause of the observed variation can be tested with the inclusion of a larger number of samples and deeper targeted sequencing in the future. This is important in order to allow us to investigate if the altered splicing events in combinations with different known mutations and risk alleles are contributing to the disease mechanisms.

In light of recent studies emphasizing mRNA splicing as an important link to diseases [33,34], we believe this type of study is an important addition to expand and build our knowledge around the complexity of splicing events that occur. These variants can affect the molecular mechanisms, the risk of mutation contributing to a disease, and functions of targeted genes in relation to neurodegenerative diseases.

## Supplementary Materials

Supplementary materials can be found at http://www.mdpi.com/1422-0067/19/9/2784/s1.
