# Supplementary material for: Insights into the Influence of Specific Splicing Events on the Structural Organization of LRRK2
Source: Int J Mol Sci. 2018 Sep 16;19(9):2784. doi: 10.3390/ijms19092784 (PMC6165039; doi:10.3390/ijms19092784)

Table of contents…………………………………………………………………….Page

Supplementary table 1 ……………………………………………………….……… II

Supplementary figure 1 ……………………………………………………..………. III

Supplementary figure 2……………………………………….…………….…….…. IV

**Supplementary Table 1.** PCR primers used in this study.

| Primer name | Sequence (5′-> 3′) | Location |
|---|---|---|
| Exon 32-33 Forward | ACCATCATAAACGAGAGCCTTAATTTC | In exon 31 |
| Exon 33 Reverse | TGCCCTTAGGGTGTTTTGGACAACCTT | In exon 34 |
| Exon 36 Reverse | CCATTCTTCCATGAGAGAATCAATG | In exon 37 |
| In Exon 39 Forward | TGATGGCAGTTTTGGATCAGTTTA | In exon 39 |
| Exon 43 Reverse | TTTAGGTAATAAAATGCGTCTCGTCAG | In exon 44 |
| In Exon 45 Reverse | TTGCAATACAAACAAGTGACAGAAT | In exon 45 |
| Exon 48 Forward | TGTGGGGAGGATGTGGCACAAAGA | In exon 47 |
| Exon 50 Reverse | GAAGATTGATGTCCCAAACGGTC | In exon 51 |

**Supplementary Figure 1.** Interactions established between the modelled WD40 domain of LRRK2 and the adjacent a-helix that was co-crystallized in the template structure. A series of specific hydrogen bonds, water-mediated bridges and hydrophobic interactions secure the association between the two proteins.

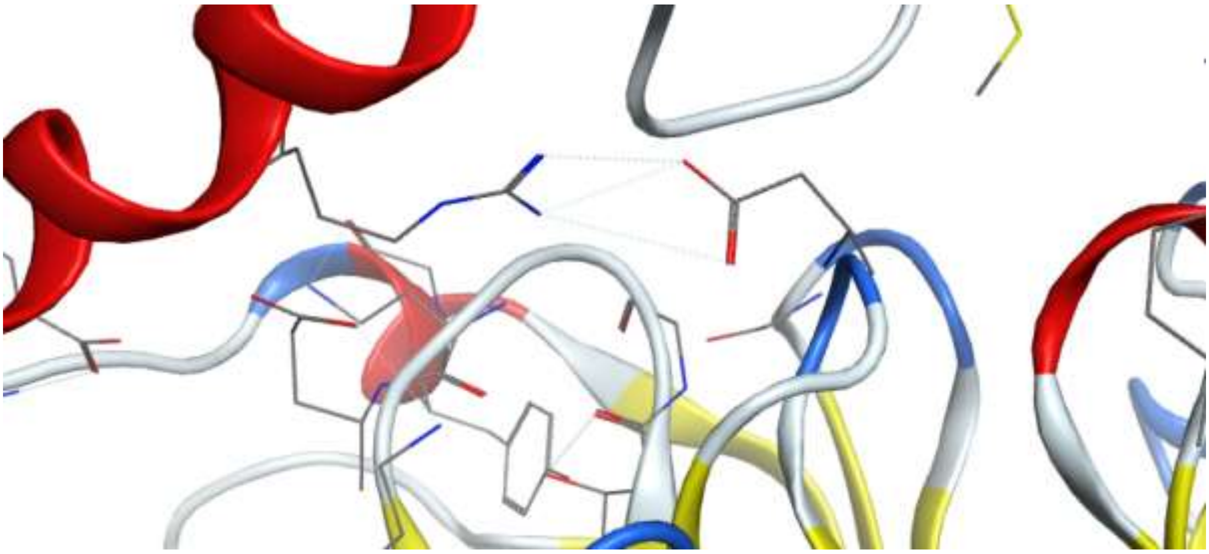

**Supplementary Figure 2.** Surface representation of the WD40 to the C-terminal a-helix of eIF3b/PRT1 interaction. The WD40 model is shown in magenta ribbon, while the sliced structure is shown in red ribbon. The C-terminal a-helix of eIF3b/PRT1 is shown in ball and stick representation in a calculated electrostatic surface. WD40's interaction domain is the one that gets removed (red ribbon) and therefore, we propose that this splicing event is a protein-protein interaction control mechanism.

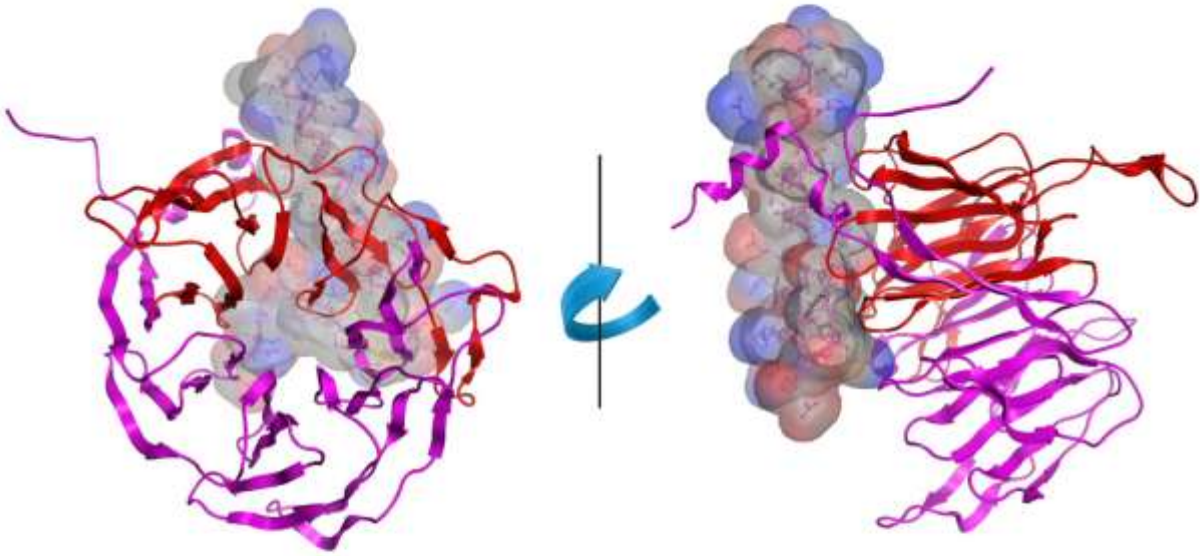

Supplement: Supplementary file 1 [file ijms-19-02784-s001.pdf]
